# The relationship between sleep status and activity of daily living: based on China Hainan centenarians cohort study

**DOI:** 10.1186/s12877-023-04480-2

**Published:** 2023-12-04

**Authors:** Shanshan Yang, Shengshu Wang, Guangdong Liu, Rongrong Li, Xuehang Li, Shimin Chen, Yali Zhao, Miao Liu, Yunxi Liu, Yao He

**Affiliations:** 1 Department of Disease Prevention and Control, First Medical Center, Chinese People’s Liberation Army General Hospital, 28 Fuxing Road, Beijing, 100853 China; 2 Institute of Geriatrics, Beijing Key Laboratory of Aging and Geriatrics, National Clinical Research Center for Geriatrics Diseases, Second Medical Center, Chinese People’s Liberation Army General Hospital, Beijing, 100853 China; 3 Sixth Medical Center, Chinese People’s Liberation Army General Hospital, Beijing, 100853 China; 4 Central Laboratory of Hainan Hospital, Chinese People’s Liberation Army General Hospital, Sanya, 572013 China; 5 Department of Statistics and Epidemiology, Graduate School, Chinese People’s Liberation Army General Hospital, Beijing, 100853 China; 6 State Key Laboratory of Kidney Diseases, Beijing, 100853 China

**Keywords:** Centenarian, Sleep duration, Sleep quality, Sleep mode, Activity of daily living

## Abstract

**Objective:**

This study, based on the China Hainan Centenarians Cohort Study (CHCCS), aims to comprehensively describe the characteristic of daytime, night and total sleep duration, sleep quality and different sleep mode of Hainan centenarians and their associations with activity of daily living (ADL) functions.

**Method:**

The baseline data of CHCCS was used. ADL function was evaluated the Bathel index, sleep quality was evaluated by Pittsburgh sleep quality index (PSQI), sleep status including daytime, night and total sleep duration as well as sleep quality and sleep mode. Multivariate logistic regression model was used to explore the association between sleep status and ADL disability and ADL moderate & severe disability.

**Results:**

A total of 994 centenarians were included in this study with the age range 100–116 years old. Compared with the centenarians who sleep 6–9 h at night and < 2 h in the daytime, the adjusted OR between sleep > 9 h at night and sleep ≥ 2 h in the daytime and ADL disability was 2.93 (95% CI: 1.02–8.44), and adjusted OR of ADL moderate & severe disability was 2.75 (95% CI: 1.56–4.83). Compared with centenarians who sleep for 7–9 h and have good sleep quality, centenarians who sleep for > 9 h and have poor sleep quality have an increased risk of ADL moderate & severe disability (OR = 3.72, 95% CI: 1.54–9.00).

**Conclusion:**

Relation between sleep duration and ADL disability was more significant compared with sleep quality in Hainan centenarians. Poor sleep quality can aggravate the relationship between sleep duration and ADL moderate & severe disability.

**Supplementary Information:**

The online version contains supplementary material available at 10.1186/s12877-023-04480-2.

## Key points

1. The median duration of daytime sleep (Q1, Q3) was 1.00 (0.50,1.50) hour, while those of night sleep and total sleep (Q1, Q3) were 8.00 (7.00,9.00) hours and 9.00 (8.00,10.50) hours in the centenarians, respectively. And 35.5% of them had poor sleep quality.

2. Compared with sleep quality, the relationship between sleep duration and activity of daily living (ADL) disability was more significant.

3.Centenarians with long sleep duration both at night and in the daytime have high risk of ADL disability and ADL moderate & severe disability.

4. Poor sleep quality could aggravate the relationship between sleep duration on ADL moderate & severe disability.

**Supplementary Information:**

The online version contains supplementary material available at 10.1186/s12877-023-04480-2.

Population aging, with the social development and progress, has become increasingly present in developed and developing countries [1]. Global average life expectancy was 72.8 years in 2019, with an increase of 9 years old than 1990. The combination of increasing life expectancy and declining fertility has exacerbated the global population aging problem. According to United Nations, the number of elderly people aged 65 or older in 2025 will account for 16% by 2050 [2]. How to cope with the rapid aging process is an important public health problem facing China at present. Older adults with impaired activity of daily living (ADL) would suffer higher health risks. The older adults maintaining better ADL function is one of the key ways to achieve a healthy aging goal. Identifying and understanding the influencing factors that may be related to the ADL function could help improve the self-care and health level of the older adults living in the community.

Sleep status can affect the ADL function of the older [3, 4], and is also an important changeable factor affecting the health. Previous studies [5, 6] have indicated that sleep status affects the risk of falls, disability, and mortality in the elderly. With the acceleration of China’s aging process, the number and proportion of the older are increasing rapidly. Previous studies have mostly categorized the elderly population aged 65 and above into a large group. There is a lack of descriptions of sleep characteristics for different age groups in the elderly population, such as those aged 80 and above, and even the centenarian group [7].

Our research team systematically reviewed the epidemiological studies of sleep disorders in the oldest-old from 2000 to 2022 [8], in this meta-analysis, 25 articles (7 Chinese and 18 English) involving 52,617 individuals aged over 80 were included. The results revealed a 28.8% prevalence rate (95% CI: 23.4%-34.9%) of sleep disorders among the elderly. Also, the review showed that the sleep problems in the oldest-old, especially the centenarians, were not paid enough attention as only one article was retrieved from the study of collecting sleep information of centenarians with structured scale. A recent study based on the oldest-old in rural China suggests [9], sleep problem will affect the ADL functions and then influence their mental health. However, the people involved in similar studies that focus on this association are mostly adults, older adults less than 80 years old, and those from nursing homes and clinical patients. The research population lack the community centenarians. Moreover, most previous sleep quality studies only included night sleep duration and Self-described sleep quality, lacking the daytime sleep duration, sleep mode and structured sleep quality scale information with few correction variables. A recent study [10] involving 1798 elderly people over the age of 90 suggested that excessive sleep duration (≥ 12 h) increases the risk of ADL disability (OR = 1.47,95%CI: 1.02–2.10). This study, however, did not provide relevant data for centenarians. Furthermore, it only evaluated the association between total sleep duration and ADL, lacking descriptions of sleep quality and other relevant information.

This study intends to use the CHCCS to explore the relationship between the sleep status (including sleep duration, sleep quality and different sleep mode and pattens) of the centenarians and their ADL disability status, and to provide basic data and scientific evidence for the health maintenance in the oldest-old and centenarian.

## Methods

### Subjects

This study is based on the baseline survey data (2014–2016) of CHCCS, a centenarian survey with the largest sample size in a single center covering the whole Hainan province. The baseline survey population includes the elderly ≥ 100 years old in 18 county-level units [11].

### Sleep duration

Average duration of night sleep and daytime sleep were recorded according to the self-report of centenarians and their families. The total sleep duration is the sum of night and day sleep duration. According to the previous study of the representative oldest-old aged 80–105 from China [12], the total sleep duration was divided into short (≤ 7 h), suitable (7–9 h) and long (> 9 h), night sleep duration was divided into the short (≤ 6 h), suitable (6–9 h) and long (> 9 h); and daytime sleep duration was divided into non-sleep, 0.5–1 h; 1–2 h and ≥ 2 h.

### Sleep mode

Sleep mode is a classification that categorizes sleep duration between night and daytime into different groups. These groups include short nighttime sleep duration (≤ 6 h), medium nighttime sleep duration (6–9 h), and long nighttime sleep duration (> 9 h). In addition, whether the duration of daytime sleep too long (≥ 2 h) is also taken into account. Based on these criteria, six sleep modes are created: short nighttime sleep duration with not too long daytime sleep duration (≤ 6 h of night sleep and < 2 h of daytime sleep), moderate nighttime sleep duration with not too long daytime sleep duration (6–9 h of night sleep and < 2 h of daytime sleep), long nighttime sleep duration with not too long daytime sleep duration (> 9 h of night sleep and < 2 h of daytime sleep), short nighttime sleep duration with long daytime sleep duration (≤ 6 h of night sleep and ≥ 2 h of daytime sleep), moderate nighttime sleep duration with long daytime sleep duration (6–9 h of night sleep and ≥ 2 h of daytime sleep), and long nighttime sleep duration with long daytime sleep duration (> 9 h of night sleep and ≥ 2 h of daytime sleep).

### Sleep quality

Pittsburgh sleep quality index (PSQI) was used to estimate the sleep quality [13], consisting of 7 contents: subjective sleep quality, sleep latency, sleep duration, habitual sleep efficiency, sleep disturbances, use of sleeping medication, daytime dysfunction. These 7 components were divided into 4 grades according to 0 ~ 3 points, and the cumulative scores of each component get the total score of PSQI ranging from 0 ~ 21 points. The higher total and each component scores, the poorer sleep quality. We categorized centenarians as either good or poor sleepers using a cut-off value (defined as PSQI ≥ 8 for poor sleep quality) [14].

The total sleep duration and sleep quality were combined and divided into the group with total sleep duration of 7–9 h and normal sleep quality, the total sleep duration 7–9 h and poor sleep quality, the total sleep duration ≤ 7 h and normal sleep quality, the total sleep duration ≤ 7 h and poor sleep quality, the total sleep duration > 9 h and normal sleep quality, and the total sleep duration > 9 h and poor sleep quality. In multivariate analysis, the merging of the original six groups into four was done to account for the significant impact caused by a small number of individuals. The four groups were total sleep duration ≤ 9 h & normal sleep quality, total sleep duration > 9 h & normal sleep quality, total sleep duration ≤ 9 h & poor sleep quality and total sleep duration > 9 h & poor sleep quality.

### Activity of daily living

ADL function was evaluated by Barthel index, containing 10 items, 8 of them involving self-care ability and 2 activity ability. The total Barthel score is 100 points and the higher score, the better ADL [15]. An overall score of 100 was defined as self-care, a score of 61–95 as mild disability (The minimum score for a single item in the Bathel index is 5 points), 41–60 as moderate disability and ≤ 40 as severe disability [16].

### Covariates

Covariates included age, gender, nationality (Han, Li and other national minority), marital status (married, widowed, divorced or single), education level (illiteracy, primary school, junior high school and above), residence type (living with family and living alone), smoking status (never, ever and current smoking), drinking status (never, ever and current drinking), exercise status (no exercise, moderate exercise and high exercise), central obesity (waist circumference ≥ 90 cm in male or ≥ 85 cm in female [17]), diet status and comorbidity. In this study, comorbidity means that more than two diseases, including diabetes, coronary heart disease, stroke, hypertension, dyslipidemia, chronic obstructive pneumonia and tumors, are determined as comorbidity if it is clearly diagnosed by the hospital [18].

### Ethics

The study has been approved by the Ethics Committee of Hainan Hospital of the General Hospital of the People’s Liberation Army (Approval No.: 301hn11201601), and the respondents have signed the informed consent form. Informed consents were obtained from authorized representatives or guardians of all illiterate participants.

## Statistical analysis

The primary data was double-entered into Epidata3.0. The Kolmogorov–Smirnov is used to test the normality of continuous variables, and the variables conforming to normal distribution or approximate normal distribution were described by mean and standard deviation, and difference was analyzed by students’ test between 2 groups and one-way ANOVA between 3 and more groups; the variables not conforming to normal distribution were described by the median and interquartile interval and the difference was analyzed by Mann–Whitney between 2 groups and Kruskal–Wallis between 3 and more groups. Categorical variables were compared between groups using Chi-square test or Fisher test as appropriate. Logistic regression models were used to explore the association between sleep status and ADL disability as well as ADL moderate & severe disability. The OR values and 95% CI were calculated. Four models to adjust different covariates were conducted. Model A was an unadjusted variable model; Model B adjusting for age and sex; Model C adjusting for age, sex, nationality, marital status, education level and residence type; Model D adjusting for age, sex, nationality, marital status, education level, residence type, smoking status, drinking status, exercise status, central obesity, diet status and comorbidity. Two-sided statistical tests were conducted, considering *p* values ≤ 0.05 as statistically significant. All analysis was conducted by SPSS 26.0.

## Result

A total of 994 centenarians, ranging from 100 to 116 years old with an average age of 102.77 ± 2.75 years and 179 men (18%), were included in this study after excluding 8 subjects with insufficient variable information in CHCCS. The median duration of daytime sleep (Q1, Q3) was 1.00 (0.50,1.50) hour, while those of night sleep and total sleep (Q1, Q3) were 8.00 (7.00,9.00) hours and 9.00 (8.00,10.50) hours in the centenarians, respectively. 64.7% of centenarians slept for fewer than 7 h or more than 9 h, while 42.2% slept for 6 h or less or more than 9 h at night. 47.2% of centenarians had the sleep mode of 6-9 h at night & < 2 h daytime. And 35.5% of them had poor sleep quality. Only 16.5% of centenarians were ADL independent; 830 centenarians scored less than 100 on the Bathel scale were determined as ADL disability and 284 (28.6%) scored less than 60 were determined as ADL moderate and severe disability.

Table 1 shows the basic characteristic of different ADL functional groups of Hainan centenarians. The distribution of residential types and exercise groups varies across ADL function groups (*P* < 0.001). The proportion of centenarians with ADL moderate & severe disability who sleep more than 9 h at night is higher at 26.8% compared to only 12.2% of independent centenarians. Additionally, the proportion of those who sleep ≥ 2 h during the daytime is higher at 22.5% among centenarians with ADL moderate & severe disability, in contrast to 18.9% among independent centenarians (Table 1).

**Table 1:** General and sleep characteristics of CHCCS

	Independent	ADL mild disability	ADL moderate and severe disability	All	*P*
	(*n* = 164)	(*n* = 546)	(*n* = 284)	(*n* = 994)	
M (Q1, Q3)					
PSQI Score (points)	6.00 (4.00,9.00)	6.00 (4.00,9.00)	6.00 (4.00,9.00)	6.00 (4.00,9.00)	0.501
Sleep duration at night (hours)	8.00 (7.00,9.00)	8.00 (7.00,9.00)	8.00 (7.00,10.00)	8.00 (7.00,9.00)	0.019
Daytime sleep duration (hours)	1.00 (0.50,1.50)	1.00 (0.50,1.50)	1.00 (0.50,1.50)	1.00 (0.50,1.50)	0.587
Total sleep duration (hours)	9.00 (8.00,10.00)	9.00 (8.00,10.25)	9.00 (8.00,10.00)	9.00 (8.00,11.00)	0.034
N (%)					
Age group					0.172
101-104ys	141 (86.0)	425 (77.8)	222 (78.2)	788 (79.3)	
105-109ys	19 (11.6)	101 (18.5)	55 (19.4)	175 (17.6)	
≥ 110ys	4 (2.4)	20 (3.7)	7 (2.5)	31 (3.1)	
Gender					< 0.001
Men	53 (32.3)	84 (15.4)	42 (14.8)	179 (18.0)	
Women	111 (67.7)	462 (84.6)	242 (85.2)	815 (82.0)	
Nationality					0.339
Han	145 (88.4)	481 (88.1)	249 (87.7)	875 (88.0)	
Li	15 (9.1)	57 (10.4)	34 (12.0)	106 (10.7)	
Others	4 (2.4)	8 (1.5)	1 (0.4)	13 (1.3)	
Marital status					0.213
Married	22 (13.4)	53 (9.7)	23 (8.1)	98 (9.9)	
Widowed	136 (82.9)	453 (83.0)	243 (85.6)	832 (83.7)	
Divorced or never married	6 (3.7)	40 (7.3)	18 (6.3)	64 (6.4)	
Education level					0.062
Illiterate	141 (86.0)	506 (92.7)	262 (92.3)	909 (91.4)	
Primary school	18 (11.0)	29 (5.3)	19 (6.7)	66 (6.6)	
Middle school or higher	5 (3.0)	11 (2.0)	3 (1.1)	19 (1.9)	
Residential type					< 0.001
Living together with families	127 (77.4)	469 (85.9)	261 (91.9)	857 (86.2)	
Living alone at home	37 (22.6)	77 (14.1)	23 (8.1)	137 (13.8)	
Smoking status					0.695
Non-smoker	146 (89.0)	505 (92.5)	260 (91.5)	911 (91.6)	
Former	11 (6.7)	25 (4.6)	16 (5.6)	52 (5.2)	
Current	7 (4.3)	16 (2.9)	8 (2.8)	31 (3.1)	
Drinking status					0.153
Non-drinker	141 (86.0)	483 (88.5)	241 (84.9)	865 (87.0)	
Former	11 (6.7)	38 (7.0)	31 (10.9)	80 (8.0)	
Current	12 (7.3)	25 (4.6)	12 (4.2)	49 (4.9)	
Physical activity					< 0.001
Low	107 (65.2)	480 (87.9)	281 (98.9)	868 (87.3)	
Medium	19 (11.6)	15 (2.7)	3 (1.1)	37 (3.7)	
High	38 (23.2)	51 (9.3)	0 (0.0)	89 (9.0)	
Central Obese					0.288
No	144 (87.8)	476 (87.2)	258 (90.8)	878 (88.3)	
Yes	20 (12.2)	70 (12.8)	26 (9.2)	116 (11.7)	
Diet regularity					0.940
Irregular	8 (4.9)	30 (5.5)	16 (5.6)	878 (88.3)	
Regular	156 (95.1)	516 (94.5)	268 (94.4)	116 (11.7)	
Comorbidity					0.695
No	132 (80.5)	430 (78.8)	219 (77.1)	781 (78.6)	
Yes	32 (19.5)	116 (21.2)	65 (22.9)	213 (21.4)	
Sleep quality (groups)					0.919
Normal	108 (65.9)	350 (64.1)	183 (64.4)	641 (64.5)	
Poor	56 (34.1)	196 (35.9)	101 (35.6)	353 (35.5)	
Sleep duration at night (groups)					0.005
6-9 h	104 (63.4)	324 (59.3)	147 (51.8)	575 (57.8)	
≤ 6 h	40 (24.4)	114 (20.9)	61 (21.5)	215 (21.6)	
> 9 h	20 (12.2)	108 (19.8)	76 (26.8)	204 (20.5)	
Daytime sleep duration (groups)					0.011
None	36 (22.0)	113 (20.7)	44 (15.5)	193 (19.4)	
< 1 h	17 (10.4)	75 (13.7)	58 (20.4)	150 (15.1)	
≥ 1 h & < 2 h	80 (48.8)	264 (48.4)	118 (41.5)	462 (46.5)	
≥ 2 h	31 (18.9)	94 (17.2)	64 (22.5)	189 (19.0)	
Total sleep duration (groups)					0.288
7-9 h	62 (37.8)	199 (36.4)	90 (31.7)	351 (35.3)	
≤ 7 h	38 (23.2)	114 (20.9)	55 (19.4)	207 (20.8)	
> 9 h	64 (39.0)	233 (42.7)	139 (48.9)	436 (43.9)	
Sleep mode					0.002
6-9 h at night & < 2 h daytime	81 (49.4)	264 (48.4)	124 (43.7)	469 (47.2)	
6-9 h at night & ≥ 2 h daytime	23 (14.0)	60 (11.0)	23 (8.1)	106 (10.7)	
≤ 6 h at night & < 2 h daytime	36 (22.0)	106 (19.4)	52 (18.3)	194 (19.5)	
≤ 6 h at night & ≥ 2 h daytime	4 (2.4)	8 (1.5)	9 (3.2)	21 (2.1)	
> 9 h at night & < 2 h daytime	16 (9.8)	82 (15.0)	44 (15.5)	142 (14.3)	
> 9 h at night & ≥ 2 h daytime	4 (2.4)	26 (4.8)	32 (11.3)	62 (6.2)	
Total sleep duration& sleep quality (6 groups)					0.481
7-9 h & Normal	43 (26.2)	126 (23.1)	55 (19.4)	224 (22.5)	
≤ 7 h & Normal	7 (4.3)	24 (4.4)	14 (4.9)	45 (4.5)	
> 9 h & Normal	58 (35.4)	200 (36.6)	114 (40.1)	372 (37.4)	
7-9 h & Poor	19 (11.6)	73 (13.4)	35 (12.3)	127 (12.8)	
≤ 7 h & Poor	31 (18.9)	90 (16.5)	41 (14.4)	162 (16.3)	
> 9 h & Poor	6 (3.7)	33 (6.0)	25 (8.8)	64 (6.4)	
Total sleep duration& sleep quality (4 groups)					0.261
≤ 9 h & normal	50 (30.5)	150 (27.5)	69 (24.3)	269 (27.0)	
> 9 h & normal	58 (35.4)	200 (36.6)	114 (40.1)	372 (37.4)	
≤ 9 h & poor	50 (30.5)	163 (29.9)	76 (26.8)	299 (29.2)	
> 9 h & poor	6 (3.7)	33 (6.0)	25 (8.8)	64 (6.4)	

Multivariate logistic regression models were used, taking the groups of different sleep duration and sleep quality as well as their combined groups were included as independent variables respectively. Tables 2 and 3 showed the adjusted ORs of association between sleep status and ADL disability as well as ADL moderate & severe disability in Hainan centenarians. The proportion of centenarians experiencing ADL disability was higher among those who had a night sleep duration of > 9 h compared to those with a sleep duration of 6–9 h (OR = 1.99, 95%CI: 1.16–3.42). Similarly, the proportion of ADL disability was higher among centenarians who had both a night sleep duration of > 9 h and a daytime sleep duration of ≥ 2 h, compared to those with a sleep duration of 6–9 h and a daytime sleep duration of < 2 h (OR = 2.93, 95%CI: 1.02–8.44). There is a significant association between night, daytime and total sleep duration and ADL moderate & severe disability. After adjustment, compared with the group with 6-9 hours of night sleep duration and 9 hours of night sleep duration and ≥2 hours of daytime sleep duration mode have higher risk for moderate & severe disability in ADL (OR=2.75, 95% CI: 1.56-4.83). Compared with centenarians with total sleep duration ≤ 9 h and good sleep quality, centenarians with total sleep duration > 9 h and poor sleep quality have a higher proportion of ADL moderate & severe disability (OR = 3.72, 95% CI: 1.54–9.00).

**Table 2:** The relationship between sleep status and ADL disability in the centenarians

	Model A	Model B	Model C	Model D
Sleep quality (groups)				
Normal	1	1	1	1
Poor	1.08 (0.76–1.53)	1.07 (0.74–1.52)	1.04 (0.72–1.49)	0.96 (0.66–1.40)
Sleep duration at night (groups)				
6-9 h	1	1	1	1
≤ 6 h	0.97 (0.65–1.45)	0.98 (0.65–1.48)	0.95 (0.63–1.45)	0.89 (0.58–1.38)
> 9 h	2.03 (1.22–3.38)	2.01 (1.20–3.35)	1.89 (1.12–3.17)	1.99 (1.16–3.42)
Daytime sleep duration (groups)				
None	1	1	1	1
< 1 h	1.79 (0.96–3.34)	1.68 (0.90–3.16)	1.62 (0.86–3.05)	1.46 (0.76–2.82)
≥ 1 h & < 2 h	1.10 (0.71–1.69)	1.08 (0.70–1.69)	1.08 (0.69–1.70)	0.94 (0.59–1.51)
≥ 2 h	1.17 (0.69–1.98)	1.15 (0.67–1.96)	1.16 (0.67–1.99)	0.97 (0.55–1.73)
Total sleep duration (groups)				
7-9 h	1	1	1	1
≤ 7 h	0.95 (0.61–1.49)	1.01 (0.64–1.59)	1.01 (0.64–1.61)	0.93 (0.58–1.51)
> 9 h	1.25 (0.85–1.83)	1.25 (0.85–1.85)	1.30 (0.88–1.92)	1.24 (0.83–1.87)
Sleep mode				
6-9 h at night & < 2 h daytime	1	1	1	1
6-9 h at night & ≥ 2 h daytime	0.75 (0.45–1.27)	0.73 (0.43–1.24)	0.75 (0.44–1.28)	0.75 (0.44–1.29)
≤ 6 h at night & < 2 h daytime	0.92 (0.59–1.41)	0.93 (0.60–1.44)	0.90 (0.58–1.41)	0.88 (0.56–1.38)
≤ 6 h at night & ≥ 2 h daytime	0.89 (0.29–2.71)	0.91 (0.29–2.82)	0.88 (0.28–2.81)	0.87 (0.27–2.76)
> 9 h at night & < 2 h daytime	1.64 (0.93–2.92)	1.58 (0.88–2.82)	1.49 (0.83–2.67)	1.49 (0.83–2.68)
> 9 h at night & ≥ 2 h daytime	3.03 (1.07–8.57)	3.13 (1.09–8.94)	2.97 (1.04–8.52)	2.93 (1.02–8.44)
Total sleep duration& sleep quality				
≤ 9 h & normal	1	1	1	1
> 9 h & normal	1.97 (1.16–3.35)	1.96 (1.15–3.34)	1.84 (1.07–3.15)	1.84 (1.05–3.22)
≤ 9 h & poor	1.17 (0.81–1.69)	1.16 (0.80–1.70)	1.12 (0.77–1.64)	1.01 (0.68–1.51)
> 9 h & poor	6.17 (0.83–46.08)	5.57 (0.74–41.85)	5.12 (0.68–38.52)	6.96 (0.86–56.53)

Model A: Crude model

Model B: Adjusted for age and sex

Model C: Adjusted for age, sex, nationality, marital status, education level and residence type

Model D: Adjusted for age, sex, nationality, marital status, education level, residence type, smoking status, drinking status, physical activity, central obese, diet regularity and comorbidity

**Table 3:** The relationship between sleep status and ADL moderate and severe disability in the centenarians

	Model A	Model B	Model C	Model D
Sleep quality (groups)				
Normal	1	1	1	1
Poor	1.00 (0.75–1.34)	1.01 (0.75–1.34)	0.98 (0.73–1.31)	0.92 (0.68–1.24)
Sleep duration at night (groups)				
6-9 h	1	1	1	1
≤ 6 h	1.15 (0.81–1.64)	1.16 (0.82–1.65)	1.13 (0.79–1.61)	1.10 (0.76–1.59)
> 9 h	1.73 (1.23–2.43)	1.72 (1.22–2.41)	1.66 (1.17–2.33)	1.67 (1.17–2.39)
Daytime sleep duration (groups)				
None	1	1	1	1
< 1 h	2.14 (1.33–3.42)	2.10 (1.31–3.37)	2.07 (1.29–3.33)	2.19 (1.33–3.59)
≥ 1 h & < 2 h	1.16 (0.78–1.73)	1.16 (0.78–1.72)	1.16 (0.78–1.74)	1.10 (0.73–1.66)
≥ 2 h	1.73 (1.10–2.72)	1.72 (1.10–2.71)	1.77 (1.12–2.80)	1.69 (1.05–2.73)
Total sleep duration (groups)				
7-9 h	1	1	1	1
≤ 7 h	1.05 (0.71–1.55)	1.06 (0.72–1.57)	1.07 (0.72–1.58)	1.04 (0.69–1.57)
> 9 h	1.36 (0.99–1.86)	1.36 (0.99–1.86)	1.39 (1.02–1.91)	1.40 (1.01–1.95)
Sleep mode				
6-9 h at night & < 2 h daytime	1	1	1	1
6-9 h at night & ≥ 2 h daytime	0.77 (0.47–1.28)	0.76 (0.46–1.26)	0.78 (0.47–1.29)	0.79 (0.47–1.33)
≤ 6 h at night & < 2 h daytime	1.02 (0.70–1.49)	1.02 (0.70–1.49)	1.00 (0.68–1.46)	0.98 (0.66–1.45)
≤ 6 h at night & ≥ 2 h daytime	2.09 (0.86–5.07)	2.09 (0.86–5.09)	2.06 (0.84–5.07)	1.96 (0.78–4.98)
> 9 h at night & < 2 h daytime	1.25 (0.83–1.88)	1.23 (0.81–1.85)	1.17 (0.77–1.78)	1.23 (0.80–1.89)
> 9 h at night & ≥ 2 h daytime	2.97 (1.73–5.09)	2.98 (1.74–5.12)	2.99 (1.73–5.17)	2.75 (1.56–4.83)
Total sleep duration& sleep quality				
≤ 9 h & normal	1	1	1	1
> 9 h & normal	1.47 (1.02–2.14)	1.46 (1.01–2.13)	1.40 (0.96–2.04)	1.36 (0.92–2.01)
≤ 9 h & poor	1.01 (0.73–1.39)	1.01 (0.73–1.39)	0.97 (0.70–1.34)	0.89 (0.64–1.25)
> 9 h & poor	3.50 (1.60–7.70)	3.44 (1.56–7.56)	3.27 (1.48–7.22)	3.72 (1.54–9.00)

Model A: Crude model

Model B: Adjusted for age and sex

Model C: Adjusted for age, sex, nationality, marital status, education level and residence type

Model D: Adjusted for age, sex, nationality, marital status, education level, residence type, smoking status, drinking status, physical activity, central obese, diet regularity and comorbidity

The results of subgroup analysis based on gender showed that the overall trend of the results is remarkably consistent with that of the female centenarians while the associations of sleep and ADL disability & moderate and severe disability were not found after adjusting for the covariates in male centenarians (Tables S1 and S2). Table S3 showed the distribution of the 7 components of PSQI and different ADL function groups and Table S4 showed the relationship between the 7 components of PSQI and ADL disability/ADL moderate and severe disability (Daytime dysfunction was found related with ADL disability/ADL moderate and severe disability).

## Discussion

This study, based on the CHCCS, described the epidemiological characteristics of sleep status (including sleep duration, sleep quality and sleep mode) among centenarians grouped by different ADL function, and explored the relationship between sleep status and ADL disability as well as moderate & severe disability. The results showed that, compared with sleep quality, the relationship between sleep duration and ADL disability was more significant. Poor sleep quality could aggravate the relationship between sleep duration and ADL moderate & severe disability.

Having a good ADL function is an indicator of the capacity of independent living in the oldest-old, On the contrary, ADL impairment can predict of the elderly’s health outcomes, including mortality [19]. The research results of the older over 85 years old in Newcastle showed that the prevalence of ADL disability was 65.4%, slightly lower than that of this study, which may be related to the younger average age of the study population [20]. From 1997 to 2006, the survey of the China Health and Nutrition Survey showed that the prevalence of ADL disability in the old population in China showed a downward trend [21]. However, the population included in the survey was the older adults over 60 years old, and the deadline for prevalence survey was more than 20 years ago. Based on the CHCCS research results from our team, the prevalence of ADL disability in Hainan centenarians was high, up to 83.5%, which has a great impact on the daily life of centenarians [22].

The results of this study showed that there was a significant association between the sleep duration and ADL disability, and the significant association between the night, daytime, total sleep duration and ADL moderate & severe disability, of these associations, the OR value of ADL moderate & severe disability with total sleep duration > 9 h and poor sleep quality group is the highest in centenarians. In the past, few studies have focused on sleep quality and the ADL function in the older adults, especially in the oldest-old, most of which focus on the population living in hospitals or nursing homes, and the indicators of sleep duration. A study based on inpatients aged over 65 years old showed that insomnia and sleep disorders could affect the ADL function [23]. Another study based on 101 old inpatients showed that there was a association between the ADL scores and sleep quality, which was different from the results of this study, and might be related to the differences in population selection and ADL scale using Katz scale [24]. However, another study focusing on stroke inpatients based on a large teaching tertiary hospital showed that there was no statistically significant association between short sleep time and impairment of ADL, which was consistent with the results of this study, but the study did not evaluate the impact of long sleep duration on ADL [25]. Another study, also based on stroke inpatients, showed that the daytime and night sleep duration were associated with poorer ADL, which was partially consistent with the this results, but the comparability was limited because the research object selected in this study was stroke inpatients [26]. Few studies focused on the relationship between sleep quality and ADL disability among the community oldest-old. Only the Chinese Longitudinal Health Longevity Survey (CLHLS) in China was retrieved and the results showed that ADL function is not associated with poor sleep quality [27], which is consistent with this study, but the study used a self-report sleep quality as the evaluation index, which is greatly affected by subjective factors. Another previous study focusing on the influencing factors of sleep quality in rural older people had shown that there is a significant association between ADL and sleep quality in rural region in Anhui Province, which might be related to the age and geographical differences of the study population [28]. We have also retrieved a study on the relationship between sleep quality and ADL of community old population in African American and the results showed that African American older adults with poor sleep quality also had more difficult daily life, such as bathing, toileting, dressing, but this study did not use the structured ADL scale to evaluate their ADL [29].

A potential explanation for the relationship between sleep status and ADL function is that poor sleep quality would lead to mental fatigue resulting in physical dysfunction, and then affect the ADL function of the elderly [30]. Poor sleep quality might have a negative impact on function. Therefore, centenarians with long total sleep duration and poor sleep quality found in this study have the highest risk of ADL moderate & severe disability, which can also be explained by this reason. The possibility of a bidirectional causal relationship between the two cannot be dismissed. Those with a moderate and severe disability have more time in bed and this generate a longer total sleep time and poor quality. The association between sleep duration and ADL in this cross-sectional study may be interactive, and the causal sequence cannot be distinguished. Future research would seek to understand the mechanism of sleep quality affecting functional status, which needs to be further verified in cohort study.

This study has some advantages. this study, based on the CHCCS baseline cross-sectional survey, described the relationship between the sleep status and the related ADL function outcomes, and analyzed the detail association between the sleep duration, sleep quality, sleep mode, sleep duration & quality group and the ADL function in Hainan centenarians, providing basic data for possible mechanism study. This study adopted a strictly designed epidemiological survey plan, strict quality control measures throughout the process, and rigorous data analysis process to ensure results were reliable and scientific.

This study has some limitations. This study is a cross-sectional survey, and it is impossible to draw a conclusion of causality. The 179 male centenarians is relatively small and the insufficient sample size could lead to the widening of the confidence interval of the OR. The subjects are all from a special long-lived island region Chinese Hainan Province and it is necessary to be cautious whether the research results are applicable to people in other regions. Although the investigator who have received unified training asked the centenarians themselves and their caregivers separately and compared them to avoid the occurrence of recalling bias as much as possible, it may not be able to avoid at all. There may be unadjusted confounding factors in the multifactor analysis of association studies.

## Conclusion

This study, based on the CHCCS, described the epidemic characteristics of sleep status including sleep duration, sleep quality and sleep mode among centenarians divided by different ADL function for the first time, explored the relationship between sleep status and ADL disability as well as ADL moderate & severe disability. The results showed that, the median duration of daytime sleep (Q1, Q3) was 1.00 (0.50,1.50) hour, while those of night sleep and total sleep (Q1, Q3) were 8.00 (7.00,9.00) hours and 9.00 (8.00,10.50) hours in the centenarians, respectively. And 35.5% of them had poor sleep quality. Compared with sleep quality, the relationship between sleep duration and ADL disability was more significant. Poor sleep quality could aggravate the relationship between sleep duration on ADL moderate & severe disability. The sleep health of centenarians should be concerned and improved.

### Supplementary Information

**Additional file 1: Table S1.** The relationship between sleep status and ADL disability in male and female centenarians. **Table S2.** The relationship between sleep status and ADL moderate and severe disability in male and female centenarians. **Table S3.** The distribution of the 7 components of PSQI and different ADL function groups. **Table S4.** The relationship between the 7 components of PSQI and ADL disability/ADL moderate and severe disability.

## Data Availability

The database of the current study is not publicly available. Prof. Yao He (yhe301@x263.net) should be contacted if someone wants to request the data from this study.
