# Supplementary material for: The relationship between sleep status and activity of daily living: based on China Hainan centenarians cohort study
Source: BMC Geriatr. 2023 Dec 4;23:796. doi: 10.1186/s12877-023-04480-2 (PMC10694970; doi:10.1186/s12877-023-04480-2)
Supplement: Supplementary file 1 — Additional file 1: Table S1. The relationship between sleep status and ADL disability in male and female centenarians. Table S2. The relationship between sleep status and ADL moderate and severe disability in male and female centenarians. Table S3. The distribution of the 7 components of PSQI and different ADL function groups. Table S4. The relationship between the 7 components of PSQI and ADL disability/ADL moderate and severe disability. [file 12877_2023_4480_MOESM1_ESM.doc]

Table S1 The relationship between sleep status and ADL disability in male and female centenarians

|  |  | Model A | Model B | Model C | Model D |
| --- | --- | --- | --- | --- | --- |
| Male | Sleep quality (groups) |  |  |  |  |
|  | Normal | 1 | 1 | 1 | 1 |
|  | Poor | 1.47 (0.74-2.93) | 1.42 (0.71-2.84) | 1.56 (0.76-3.22) | 1.47 (0.68-3.14) |
|  | Sleep duration at night (groups) | |  |  |  |
|  | 6-9h | 1 | 1 | 1 | 1 |
|  | ≤6h | 2.05 (0.89-4.73) | 2.08 (0.90-4.83) | 1.89 (0.79-4.49) | 1.81 (0.72-4.55) |
|  | ＞9h | 2.33 (0.88-6.18) | 2.42 (0.91-6.44) | 1.94 (0.71-5.3) | 2.25 (0.78-6.49) |
|  | Daytime sleep duration (groups) | |  |  |  |
|  | None | 1 | 1 | 1 | 1 |
|  | <1h | 2.13 (0.59-7.62) | 2.16 (0.6-7.79) | 1.95 (0.53-7.22) | 1.90 (0.48-7.58) |
|  | ≥1h & <2h | 0.73 (0.28-1.93) | 0.74 (0.28-1.95) | 0.70 (0.25-1.97) | 0.71 (0.23-2.17) |
|  | ≥2h | 1.39 (0.61-3.16) | 1.45 (0.64-3.32) | 1.48 (0.63-3.48) | 1.65 (0.65-4.19) |
|  | Total sleep duration (groups) |  |  |  |  |
|  | 7-9h | 1 | 1 | 1 | 1 |
|  | ≤7h | 2.10 (0.85-5.17) | 2.06 (0.83-5.09) | 2.09 (0.82-5.35) | 2.06 (0.77-5.51) |
|  | ＞9h | 1.14 (0.55-2.35) | 1.11 (0.53-2.29) | 1.10 (0.51-2.36) | 1.12 (0.50-2.48) |
|  | Sleep mode |  |  |  |  |
|  | 6-9h at night & <2h daytime | 1 | 1 | 1 | 1 |
|  | 6-9h at night & ≥2h daytime | 0.50 (0.17-1.46) | 0.47 (0.16-1.39) | 0.49 (0.16-1.50) | 0.56 (0.17-1.78) |
|  | ≤6h at night & <2h daytime | 2.67 (1.01-7.08) | 2.66 (1.00-7.09) | 2.51 (0.92-6.90) | 2.62 (0.92-7.51) |
|  | ≤6h at night & ≥2h daytime | 0.17 (0.02-1.67) | 0.18 (0.02-1.85) | 0.17 (0.02-1.81) | 0.10 (0.01-1.82) |
|  | >9h at night & <6h daytime | 1.88 (0.57-6.14) | 1.95 (0.59-6.43) | 1.73 (0.52-5.79) | 1.97 (0.57-6.83) |
|  | >9h at night & <7h daytime | 2.50 (0.52-12.14) | 2.52 (0.52-12.3) | 1.90 (0.38-9.67) | 1.81 (0.34-9.67) |
|  | Total sleep duration& sleep quality | |  |  |  |
|  | ≤9h & normal | 1 | 1 | 1 | 1 |
|  | ＞9h & normal | 2.73 (0.94-7.88) | 2.91 (1.00-8.45) | 2.41 (0.81-7.2) | 2.53 (0.82-7.87) |
|  | ≤9h & poor | 1.86 (0.90-3.84) | 1.84 (0.88-3.81) | 1.95 (0.91-4.18) | 1.78 (0.80-3.98) |
|  | ＞9h & poor | 1.19 (0.10-13.60) | 0.84 (0.07-10.38) | 0.73 (0.06-9.57) | 1.32 (0.08-21.26) |
| Female | Sleep quality (groups) |  |  |  |  |
|  | Normal | 1 | 1 | 1 | 1 |
|  | Poor | 0.97 (0.64-1.47) | 0.95 (0.63-1.45) | 0.92 (0.60-1.41) | 0.88 (0.56-1.38) |
|  | Sleep duration at night (groups) | |  |  |  |
|  | 6-9h | 1 | 1 | 1 | 1 |
|  | ≤6h | 0.75 (0.47-1.20) | 0.75 (0.47-1.21) | 0.76 (0.47-1.21) | 0.70 (0.42-1.16) |
|  | ＞9h | 1.86 (1.02-3.41) | 1.87 (1.02-3.43) | 1.86 (1.01-3.42) | 1.86 (1.00-3.52) |
|  | Daytime sleep duration (groups) | |  |  |  |
|  | None | 1 | 1 | 1 | 1 |
|  | <1h | 1.57 (0.76-3.23) | 1.55 (0.75-3.21) | 1.49 (0.72-3.09) | 1.48 (0.69-3.18) |
|  | ≥1h & <2h | 0.98 (0.58-1.66) | 0.97 (0.58-1.65) | 0.95 (0.56-1.61) | 0.84 (0.48-1.48) |
|  | ≥2h | 1.36 (0.7-2.63) | 1.38 (0.71-2.67) | 1.34 (0.68-2.63) | 1.16 (0.57-2.35) |
|  | Total sleep duration (groups) |  |  |  |  |
|  | 7-9h | 1 | 1 | 1 | 1 |
|  | ≤7h | 0.76 (0.45-1.27) | 0.76 (0.45-1.29) | 0.78 (0.46-1.31) | 0.71 (0.41-1.24) |
|  | ＞9h | 1.29 (0.81-2.06) | 1.31 (0.83-2.09) | 1.33 (0.83-2.13) | 1.24 (0.76-2.03) |
|  | Sleep mode |  |  |  |  |
|  | 6-9h at night & <2h daytime | 1 | 1 | 1 | 1 |
|  | 6-9h at night & ≥2h daytime | 0.78 (0.42-1.46) | 0.81 (0.43-1.52) | 0.81 (0.43-1.53) | 0.80 (0.42-1.51) |
|  | ≤6h at night & <2h daytime | 0.65 (0.40-1.07) | 0.66 (0.40-1.09) | 0.67 (0.40-1.10) | 0.65 (0.39-1.07) |
|  | ≤6h at night & ≥2h daytime | 2.49 (0.32-19.17) | 2.52 (0.33-19.45) | 2.36 (0.31-18.28) | 2.28 (0.29-17.75) |
|  | >9h at night & <6h daytime | 1.44 (0.74-2.80) | 1.46 (0.75-2.84) | 1.44 (0.74-2.82) | 1.40 (0.71-2.74) |
|  | >9h at night & <7h daytime | 3.73 (0.88-15.83) | 3.75 (0.88-15.93) | 3.81 (0.89-16.26) | 3.55 (0.83-15.15) |
|  | Total sleep duration& sleep quality | |  |  |  |
|  | ≤9h & normal | 1 | 1 | 1 | 1 |
|  | ＞9h & normal | 1.71 (0.92-3.18) | 1.71 (0.92-3.17) | 1.68 (0.9-3.14) | 1.67 (0.87-3.21) |
|  | ≤9h & poor | 0.99 (0.64-1.54) | 0.98 (0.63-1.52) | 0.95 (0.61-1.48) | 0.90 (0.56-1.44) |
|  | ＞9h & poor | (-) | (-) | (-) | (-) |

Model A: Crude model;

Model B: Adjusted for age;

Model C: Adjusted for age, nationality, marital status, education level and residence type;

Model D: Adjusted for age, nationality, marital status, education level, residence type, smoking status, drinking status, physical activity, central obese, diet regularity and comorbidity.

Table S2 The relationship between sleep status and ADL moderate and severe disability in male and female centenarians

|  |  | Model A | Model B | Model C | Model D |
| --- | --- | --- | --- | --- | --- |
| Male | Sleep quality (groups) |  |  |  |  |
|  | Normal | 1 | 1 | 1 | 1 |
|  | Poor | 1.11 (0.54-2.26) | 1.11 (0.54-2.27) | 1.16 (0.56-2.44) | 0.91 (0.40-2.06) |
|  | Sleep duration at night (groups) | |  |  |  |
|  | 6-9h | 1 | 1 | 1 | 1 |
|  | ≤6h | 1.36 (0.59-3.12) | 1.36 (0.59-3.12) | 1.28 (0.54-3.03) | 1.04 (0.39-2.75) |
|  | ＞9h | 1.56 (0.63-3.87) | 1.56 (0.63-3.88) | 1.31 (0.52-3.35) | 1.37 (0.48-3.91) |
|  | Daytime sleep duration (groups) | |  |  |  |
|  | None | 1 | 1 | 1 | 1 |
|  | <1h | 1.03 (0.39-2.75) | 1.03 (0.38-2.74) | 1.11 (0.4-3.04) | 1.02 (0.33-3.17) |
|  | ≥1h & <2h | 1.79 (0.58-5.50) | 1.79 (0.58-5.50) | 1.90 (0.59-6.10) | 1.66 (0.44-6.30) |
|  | ≥2h | 4.16 (1.27-13.58) | 4.15 (1.27-13.57) | 4.14 (1.23-13.92) | 4.29 (1.09-16.96) |
|  | Total sleep duration (groups) |  |  |  |  |
|  | 7-9h | 1 | 1 | 1 | 1 |
|  | ≤7h | 1.09 (0.44-2.68) | 1.09 (0.44-2.69) | 1.15 (0.45-2.94) | 1.01 (0.35-2.95) |
|  | ＞9h | 1.02 (0.46-2.28) | 1.02 (0.45-2.29) | 1.10 (0.47-2.56) | 1.09 (0.44-2.72) |
|  | Sleep mode |  |  |  |  |
|  | 6-9h at night & <2h daytime | 1 | 1 | 1 | 1 |
|  | 6-9h at night & ≥2h daytime | 0.50 (0.11-2.39) | 0.50 (0.11-2.39) | 0.54 (0.11-2.65) | 0.66 (0.13-3.44) |
|  | ≤6h at night & <2h daytime | 1.25 (0.52-3.00) | 1.25 (0.52-3.00) | 1.18 (0.48-2.93) | 1.06 (0.38-2.95) |
|  | ≤6h at night & ≥2h daytime | 1.17 (0.12-11.84) | 1.17 (0.11-11.91) | 1.38 (0.13-15.05) | 0.62 (0.03-13.03) |
|  | >9h at night & <6h daytime | 0.66 (0.17-2.48) | 0.66 (0.17-2.48) | 0.58 (0.15-2.24) | 0.74 (0.17-3.28) |
|  | >9h at night & <7h daytime | 3.50 (1.02-12.05) | 3.50 (1.02-12.05) | 2.88 (0.80-10.35) | 2.30 (0.56-9.50) |
|  | Total sleep duration& sleep quality | |  |  |  |
|  | ≤9h & normal | 1 | 1 | 1 | 1 |
|  | ＞9h & normal | 1.92 (0.74-4.99) | 1.94 (0.75-5.07) | 1.70 (0.63-4.58) | 1.50 (0.50-4.49) |
|  | ≤9h & poor | 1.41 (0.65-3.07) | 1.41 (0.65-3.07) | 1.45 (0.65-3.24) | 1.04 (0.43-2.50) |
|  | ＞9h & poor | 0 (0-.) | 0 (0-.) | 0 (0-.) | 0 (0-.) |
| Female | Sleep quality (groups) |  |  |  |  |
|  | Normal | 1 | 1 | 1 | 1 |
|  | Poor | 0.99 (0.72-1.35) | 0.99 (0.72-1.35) | 0.96 (0.70-1.31) | 0.92 (0.66-1.27) |
|  | Sleep duration at night (groups) | |  |  |  |
|  | 6-9h | 1 | 1 | 1 | 1 |
|  | ≤6h | 1.12 (0.76-1.65) | 1.12 (0.76-1.65) | 1.10 (0.74-1.62) | 1.07 (0.71-1.61) |
|  | ＞9h | 1.74 (1.20-2.51) | 1.74 (1.20-2.51) | 1.71 (1.18-2.48) | 1.67 (1.14-2.47) |
|  | Daytime sleep duration (groups) | |  |  |  |
|  | None | 1 | 1 | 1 | 1 |
|  | <1h | 1.18 (0.77-1.82) | 1.18 (0.77-1.82) | 1.20 (0.78-1.86) | 1.20 (0.76-1.89) |
|  | ≥1h & <2h | 1.71 (1.04-2.80) | 1.71 (1.04-2.80) | 1.80 (1.09-2.97) | 1.80 (1.06-3.05) |
|  | ≥2h | 1.87 (1.12-3.13) | 1.87 (1.12-3.13) | 1.88 (1.12-3.16) | 2.16 (1.25-3.74) |
|  | Total sleep duration (groups) |  |  |  |  |
|  | 7-9h | 1 | 1 | 1 | 1 |
|  | ≤7h | 1.05 (0.68-1.62) | 1.05 (0.68-1.62) | 1.05 (0.68-1.62) | 1.05 (0.67-1.65) |
|  | ＞9h | 1.43 (1.01-2.00) | 1.43 (1.02-2.01) | 1.46 (1.04-2.06) | 1.49 (1.04-2.13) |
|  | Sleep mode |  |  |  |  |
|  | 6-9h at night & <2h daytime | 1 | 1 | 1 | 1 |
|  | 6-9h at night & ≥2h daytime | 0.81 (0.47-1.38) | 0.81 (0.47-1.38) | 0.82 (0.48-1.42) | 0.84 (0.48-1.48) |
|  | ≤6h at night & <2h daytime | 0.97 (0.64-1.48) | 0.97 (0.64-1.48) | 0.96 (0.63-1.46) | 0.95 (0.61-1.48) |
|  | ≤6h at night & ≥2h daytime | 2.35 (0.88-6.26) | 2.35 (0.88-6.26) | 2.30 (0.86-6.16) | 2.00 (0.73-5.50) |
|  | >9h at night & <6h daytime | 1.32 (0.85-2.05) | 1.32 (0.85-2.05) | 1.27 (0.82-1.98) | 1.28 (0.81-2.03) |
|  | >9h at night & <7h daytime | 2.87 (1.57-5.21) | 2.87 (1.57-5.21) | 3.01 (1.64-5.53) | 2.74 (1.45-5.15) |
|  | Total sleep duration& sleep quality | |  |  |  |
|  | ≤9h & normal | 1 | 1 | 1 | 1 |
|  | ＞9h & normal | 1.39 (0.93-2.09) | 1.39 (0.93-2.09) | 1.36 (0.91-2.05) | 1.31 (0.86-2.01) |
|  | ≤9h & poor | 0.94 (0.66-1.34) | 0.94 (0.66-1.34) | 0.91 (0.63-1.30) | 0.86 (0.59-1.24) |
|  | ＞9h & poor | 4.33 (1.84-10.21) | 4.33 (1.84-10.20) | 4.07 (1.72-9.62) | 4.02 (1.58-10.27) |

Model A: Crude model;

Model B: Adjusted for age;

Model C: Adjusted for age, nationality, marital status, education level and residence type;

Model D: Adjusted for age, nationality, marital status, education level, residence type, smoking status, drinking status, physical activity, central obese, diet regularity and comorbidity.

Table S3 the distribution of the 7 components of PSQI and different ADL function groups

|  | ADL Independent | ADL dependent | P |
| --- | --- | --- | --- |
|  | (n=164) | (n=830) |  |
| Subjective sleep quality |  |  | 0.283 |
| 0 | 8 (4.9) | 22 (2.7) |  |
| 1 | 54 (32.9) | 240 (28.9) |  |
| 2 | 85 (51.8) | 470 (56.6) |  |
| 3 | 17 (10.4) | 98 (11.8) |  |
| Sleep latency |  |  | 0.296 |
| 0 | 19 (11.6) | 128 (15.4) |  |
| 1 | 73 (44.5) | 372 (44.8) |  |
| 2 | 40 (24.4) | 210 (25.3) |  |
| 3 | 32 (19.5) | 120 (14.5) |  |
| Sleep duration |  |  | 0.083 |
| 0 | 100 (61.0) | 547 (65.9) |  |
| 1 | 34 (20.7) | 191 (23.0) |  |
| 2 | 17 (10.4) | 54 (6.5) |  |
| 3 | 13 (7.9) | 38 (4.6) |  |
| Habitual sleep efficiency |  |  | 0.228 |
| 0 | 65 (39.6) | 393 (47.3) |  |
| 1 | 42 (25.6) | 165 (19.9) |  |
| 2 | 24 (14.6) | 124 (14.9) |  |
| 3 | 33 (20.1) | 148 (17.8) |  |
| Sleep disturbances |  |  | 0.095 |
| 0 | 11 (6.7) | 57 (6.9) |  |
| 1 | 121 (73.8) | 533 (64.2) |  |
| 2 | 30 (18.3) | 227 (27.3) |  |
| 3 | 2 (1.2) | 13 (1.6) |  |
| Use of sleeping medication | |  | 0.423 |
| 0 | 164 (100) | 816 (98.3) |  |
| 1 | 0 (0) | 10 (1.2) |  |
| 2 | 0 (0) | 3 (0.4) |  |
| 3 | 0 (0) | 1 (0.1) |  |
| Daytime dysfunction |  |  | 0.004 |
| 0 | 105 (64) | 461 (55.5) |  |
| 1 | 32 (19.5) | 131 (15.8) |  |
| 2 | 21 (12.8) | 137 (16.5) |  |
| 3 | 6 (3.7) | 101 (12.2) |  |

Table S4 The relationship between the 7 components of PSQI and ADL disability/ADL moderate and severe disability

|  | ADL disability | ADL moderate and severe disability |
| --- | --- | --- |
| Subjective sleep quality |  |  |
| 0 | 1 | 1 |
| 1 | 1.30 (0.49-3.47) | 1.07 (0.41-2.76) |
| 2 | 1.53 (0.59-3.98) | 1.25 (0.49-3.16) |
| 3 | 1.45 (0.49-4.26) | 1.32 (0.49-3.57) |
| Sleep latency |  |  |
| 0 | 1 | 1 |
| 1 | 0.66 (0.36-1.19) | 1.13 (0.73-1.75) |
| 2 | 0.69 (0.37-1.31) | 1.07 (0.66-1.72) |
| 3 | 0.41 (0.21-1.80) | 0.96 (0.57-1.64) |
| Sleep duration |  |  |
| 0 | 1 | 1 |
| 1 | 0.94 (0.60-1.47) | 0.82 (0.58-1.17) |
| 2 | 0.55 (0.29-1.04) | 0.85 (0.48-1.51) |
| 3 | 0.42 (0.21-1.05) | 0.81 (0.42-1.56) |
| Habitual sleep efficiency |  |  |
| 0 | 1 | 1 |
| 1 | 0.57 (0.36-0.91) | 0.95 (0.65-1.39) |
| 2 | 0.82 (0.47-1.43) | 0.82 (0.53-1.26) |
| 3 | 0.58 (0.35-1.95) | 0.71 (0.47-1.07) |
| Sleep disturbances |  |  |
| 0 | 1 | 1 |
| 1 | 0.78 (0.38-1.60) | 1.55 (0.79-3.02) |
| 2 | 1.22 (0.55-2.71) | 2.39 (1.19-4.80) |
| 3 | 0.94 (0.17-5.18) | 2.50 (0.71-8.73) |
| Use of sleeping medication | |  |
| 0 | 1 | 1 |
| 1 | (-) | 2.08 (0.53-8.16) |
| 2 | (-) | 1.19 (0.1-14.07) |
| 3 | (-) | (-) |
| Daytime dysfunction |  |  |
| 0 | 1 | 1 |
| 1 | 0.94 (0.59-1.52) | 0.54 (0.35-0.84) |
| 2 | 1.26 (0.73-2.16) | 0.75 (0.50-1.13) |
| 3 | 3.75 (1.54-9.11) | 1.62 (1.03-2.54) |

Adjusted for age, gender, nationality, marital status, education level, residence type, smoking status, drinking status, physical activity, central obese, diet regularity and comorbidity.
